# Green Coffee Extract Microencapsulated: Physicochemical Characteristics, Stability, Bioaccessibility, and Sensory Acceptability through Dairy Beverage Consumption

**DOI:** 10.3390/ijerph192013221

**Published:** 2022-10-14

**Authors:** Laísa Bernabé do Carmo, Daiane Bonizioli Benincá, Mariana Grancieri, Lucélia Vieira Pereira, Tarcísio Lima Filho, Sérgio Henriques Saraiva, Pollyanna Ibrahim Silva, Daniela da Silva Oliveira, André Gustavo Vasconcelos Costa

**Affiliations:** 1 Postgraduate Program in Food Science and Technology, Centre of Agricultural and Engineering Sciences, Federal University of Espirito Santo, Alegre 29500-000, ES, Brazil; 2 Centre of Exact, Natural and Health Sciences, Department of Pharmacy and Nutrition, Federal University of Espirito Santo, Alto Universitário, Guararema, Alegre 29500-000, ES, Brazil; 3 Department of Nutrition and Health, Federal University of Viçosa, Viçosa 36570-900, MG, Brazil; 4 Department of Food Engineering, Centre of Agricultural and Engineering Sciences, Federal University of Espirito Santo, Alegre 29500-000, ES, Brazil

**Keywords:** *Coffea canephora*, bioactive compounds, spray-drying, freeze-drying, polydextrose, inulin

## Abstract

This study aimed to investigate the effect of spray drying (SD) and freeze-drying (FD) on the microencapsulation of green coffee extracts by using polydextrose (PD) and inulin (IN) as encapsulating agents and their physicochemical, bioactive compounds’ stability, phenolic compounds’ bioaccessibility after digestion, and sensory effects in unfermented dairy beverages. The extract encapsulated with IN by FD had lower moisture content, water activity, and hygroscopicity, while particles encapsulated by SD exhibited a spherical shape and the structure of the FD products was irregular. No difference was observed in phenolic compounds’ bioaccessibility. Dairy beverages with added encapsulated extracts had higher total phenolic content and antioxidant activity. Microencapsulation allowed a controlled release of the bioactive compounds with an increase in the content of caffeine, chlorogenic acid, and trigonelline during storage. The dairy beverage with added extract encapsulated with IN by FD had the highest scores of acceptability regarding the overall impression and purchase intent.

## 1. Introduction

Green coffee beans are the unroasted seeds of *Coffea* fruits, and are rich in bioactive compounds such as chlorogenic acid, caffeine, and trigonelline [1]. Their chemical composition depends on intrinsic factors such as the species and degree of maturation, as well as on extrinsic factors such as the climate and soil composition. Green coffee beans of the *Coffea canephora* species have a higher content of chlorogenic acid and caffeine than *Coffea arabica*, which are the main bioactive compounds that contribute to the high antioxidant activity of green coffee [2]. 

Studies suggest that the consumption of green coffee has a beneficial effect on insulin resistance, reducing the risk of diabetes and cardiovascular diseases, in addition to having anticarcinogenic effects and even activity against COVID-19 [1,3]. These beneficial properties of the constituents of green coffee have contributed to an increased interest in their incorporation into food products [4,5]. A significant increase in the products’ phenolic content and antioxidant capacity, in addition to antioxidant activity against lipid oxidation, is the result, which improves the quality and stability of the products [6]. 

However, natural antioxidants are sensitive to heat and light and are easily oxidized, which limits their application in the food industry. One way to minimize this problem is the use of encapsulation techniques, including spray drying (SD) and freeze-drying (FD), using encapsulating materials. These technologies allow the substances of interest to be incorporated into a coating matrix, which delays the degradation processes, controls their release, and improves their stability [7]. The atomization and lyophilization drying techniques are well-established and widely used for the encapsulation of various pharmaceutical substances, microorganisms, polyphenols, vitamins, minerals, and dyes [7], allowing the best use of these compounds in food products.

Dairy beverages are smooth and versatile drinks that have high nutritional value owing to their protein, lactose, mineral, and vitamin content. In addition, they are a major alternative to whey. Consumer demand for healthier, safer, and more practical foods has contributed to the increase in the production of dairy beverages in recent years [8]. In this context, the main objective of this study was to investigate the effect of the SD and FD techniques on the microencapsulation of green coffee extracts by using two encapsulating agents and including the extracts in unfermented dairy beverages to evaluate their effects on physicochemical characteristics, stability of bioactive compounds, phenolic compounds bioaccessibility after in vitro digestibility, and sensory acceptance. This work hypothesizes that the microencapsulation of green coffee, in both encapsulating techniques and agents, will improve the antioxidant activity, physicochemical characteristics, and acceptability of unfermented dairy beverages, in addition to providing better bioaccessibility of phenolic compounds after in vitro digestibility.

## 2. Materials and Methods

Green coffee beans of the Conilon variety (*Coffea canephora*) were purchased from the Encosta do Caparaó property, located in Castelo-ES, Brazil (latitude 20°57′ S, longitude 41°31′ E, and altitude 110 m), and were harvested in the 2017/2018 season.

### 2.1. Preparation of the Aqueous Green Coffee Extract

The coffee beans were ground in a Willy SL-31 (Solab, Piracicaba, Brazil) knife mill and sieved to obtain particles ranging from 1 mm to 1.18 mm. The aqueous green coffee extract (GCE) was obtained by performing a three-stage countercurrent leaching process. In each stage, 20 g of ground green coffee was used to extract the soluble solids with 100 mL of water at 100 °C and atmospheric pressure. The GCE samples were collected after obtaining a concentration of 10 °Bx. Subsequently, the extracts were centrifuged at 2260× *g* for 5 min. 

### 2.2. Microencapsulation Procedures 

The GCE was microencapsulated using polydextrose (PD) and inulin (IN) as encapsulating agents, both at a concentration of 30% and previously diluted with distilled water. Predetermined amounts of GCE and encapsulating agents were mixed at a 1:2 volume ratio and homogenized in a magnetic stirrer for 10 min. The total soluble solids content (°Brix) was determined using a digital refractometer and the samples were subjected to the spray drying (SD) and freeze-drying (FD) processes.

The SD was performed using a mini spray dryer ADL 311S (Yamato Scientific Co., Tokyo, Japan) with a maximum compressed air pressure of 0.1 MPa, feed flow rate of 2 mL/min, drying airflow rate of 0.21 m³/min, and inlet air temperature of 150 °C. For FD, the samples were first frozen at −20 °C for 48 h and subsequently freeze-dried in an L101 benchtop freeze-dryer (Liotop, São Carlos, Brazil) at −50 °C and approximately 200 μmHg for five days. The microencapsulated extracts were stored in polyethylene-laminated containers at −80 °C until the moment of analysis. 

### 2.3. Characterization of the Encapsulated Extracts

The moisture and pH of the encapsulated extracts were determined according to the AOAC methods [9]. Water activity (Aw) was determined by direct reading on a LabMaster water activity meter (Novasina, Lachen, Switzerland). Hygroscopicity was determined by placing 2 g of the samples in desiccators containing saturated NaCl solutions (75% relative humidity). After one week, the samples were weighed and the hygroscopicity was calculated [10].

Solubility was determined by diluting 0.5 g of the samples in 50 mL of distilled water, with shaking at 100 rpm for 30 min and subsequent centrifugation at 1570× *g* for 5 min. Subsequently, 25 mL of the supernatant was dried in an oven at 105 °C until constant weight [11]. 

#### 2.3.1. Total Phenolic Compounds 

Total phenolic content was determined using the method of Singleton & Jr. [12] with adaptations. A volume of 0.6 mL of the encapsulated extracts (diluted with distilled water at 0.1:10 volume ratio) was mixed with 3 mL of Folin–Ciocalteu reagent (diluted with distilled water at 1:10 volume ratio). After 3 min, 2.4 mL of a saturated solution of Na_2_CO_3_ were added. The absorbance of the samples was measured in a spectrophotometer (Termo Fisher Scientific, Waltham, MA, USA) at 760 nm after 60 min of incubation at room temperature and in the dark. The results were expressed as mg gallic acid equivalent (GAE)/g of sample. The % of remaining was calculated considering the Time 0 as standard.

#### 2.3.2. Antioxidant Activity by ABTS Radical Assay 

The radical was prepared by adding 7 mM aqueous solution of ABTS (2,2′-azino-bis (3-ethylbenzothiazoline-6-sulfonic acid)) to a 2.45 mM solution of potassium persulfate. The mixture was stored for 16 h in the dark under refrigeration and diluted with ethanol to an absorbance of 0.700 ± 0.05 at 734 nm. Then, 0.5 mL of the encapsulated extracts (diluted in water at 0.1:50 volume ratio) were added to 3.5 mL of the diluted ABTS•+ radical. At 6 min into the reaction, absorbance readings were performed in a spectrophotometer (Termo Fisher Scientific, USA) at 734 nm and in the absence of light. Trolox was used as the standard to prepare a calibration curve and the results were expressed as μmol of Trolox/g of sample. The % remaining was calculated considering the Time 0 as standard.

#### 2.3.3. Antioxidant Activity by DPPH Radical Assay 

Antioxidant capacity was also determined using the DPPH (2,2-diphenyl-1-picrylhydrazyl) radical [13]. A 1 mM ethanolic solution of DPPH was diluted with ethanol to an absorbance of 0.700 ± 0.05 at 515 nm. Subsequently, 0.5 mL of the encapsulated extracts (diluted with water at 0.1:50 volume ratio) were added to 3.5 mL of the diluted DPPH radical solution. After mixing, absorbance was measured in a spectrophotometer (Thermo Fisher Scientific, USA) at 515 nm. The measurement was performed at 10 min of reaction for the samples of extracts encapsulated with IN and PD by FD, at 20 min for the extracts encapsulated with IN by SD, and at 25 min for the extracts encapsulated with PD by SD. The results were expressed as μmol of Trolox/g of sample. The % remaining was calculated considering the Time 0 as standard.

#### 2.3.4. Determination of Caffeine, Chlorogenic Acid, and Trigonelline 

The simultaneous determination of caffeine, chlorogenic acid, and trigonelline was performed using an Acquity UPLC chromatography system (Waters Corporation, Milford, MA, USA), using methodology described by [14,15]. In brief, 2 μL of the encapsulated extracts (diluted at 0.1:10 volume ratio) were injected into a C18 reversed-phase column (21 × 50 mm, 1.7 μm) at 40 °C. The mobile phase consisted of a mixture of methanol, water, and acetic acid (20:80:1 *v*/*v*/*v*). The setting was as follows: flow rate of 0.1 mL/min, isocratic elution, run-time of 10 min, and detection at 272 nm. The samples and the mobile phase were filtered through 0.22 μm membranes before analysis. 

The identification of the compounds was performed by comparing the retention times of the samples with those of the commercial standards of caffeine (Proquímicos, Brazil), chlorogenic acid (Sigma-Aldrich, Darmstadt, Germany), and trigonelline (Sigma-Aldrich, Germany). The quantification was based on the calibration curves using the caffeine (0.75–150 mg/L; R^2^ = 1), chlorogenic acid, and trigonelline (0.75–150 mg/L; R^2^ = 0.99) standards.

#### 2.3.5. Scanning Electron Microscopy (SEM) 

The microstructure of the encapsulated extracts was evaluated using an adaptation of the method proposed by [16]. The samples were placed on an adhesive tape adhered to a metal base and coated with a thin layer of gold under vacuum conditions. Particle size and structure were analyzed using a JSM-6610 LV scanning electron microscope (JEOL Ltd., Tokyo, Japan), under 100-, 400-, 3000-, and 8000-fold magnification.

### 2.4. Incorporation into Dairy Beverages 

Each portion (200 mL) of the cappuccino-flavored unfermented dairy beverages contained a mixture of 120 mL of pasteurized milk and 80 mL of whey, which constituted the dairy base. The formulation also included 14 g of sucrose, 4 g of powdered chocolate, 2 g of instant coffee, 0.4 g of stabilizer gelatin, and 0.1 g of cinnamon powder. The beverages of the test groups contained 10 g of encapsulated extracts.

Then, five beverages were prepared: the control beverage (CT), without encapsulated extract; a dairy beverage with the addition of extract encapsulated with polydextrose by spray drying (PD-SD); a dairy beverage with the extract encapsulated with inulin by spray drying (IN-SD); a dairy beverage with the extract encapsulated with polydextrose by freeze-drying (PD-FD); and a dairy beverage with the extract encapsulated with inulin by freeze-drying (IN-FD).

During the processing of the beverages, whey was filtered and heated (70 °C/30 min) and the mixture of whey, pasteurized milk, sugar, and stabilizer was subsequently pasteurized with stirring (65 °C/30 min). The mixture was cooled (40 °C) and the chocolate powder, instant coffee, cinnamon, and encapsulated extracts were added. The obtained beverage was transferred to opaque polyethylene containers and kept refrigerated (2 to 10 °C).

### 2.5. Stability of Compounds in the Dairy Beverages during Storage 

The stability of the compounds in the unfermented dairy beverages was analyzed over seven days of storage (1 week). The extracts used in the beverages were obtained using a method adapted from [5]. In brief, 1 mL of each beverage was submitted to extraction with 20 mL of phosphate buffered saline (PBS) buffer (pH 7.4) for one hour with subsequent centrifugation (1150× *g* for 5 min). The supernatant was separated and the residue extracted again with 20 mL of PBS buffer. Finally, the supernatants were combined, added to 1 mL of acetonitrile, and centrifuged (5284× *g* for 15 min).

The quantification of caffeine, chlorogenic acid, and trigonelline in the extracts of the dairy beverages was performed at 0, 24, 48, 72, 96, 120, 144 and 168 h (daily for 1 week), following the method described in Section 2.3.4 above, to verify the stability of the compounds over 1 week.

The content of total phenolics and antioxidant activity of the extracts used in the dairy beverages were analyzed at 0 and 168 h (as Section 2.3.1, Section 2.3.2 and Section 2.3.3 above). In the ABTS and DPPH radical assays, the extracts were diluted with PBS buffer (1:25 volume ratio) and PBS buffer (1:10 volume ratio), respectively, and the absorbance was measured at 10 min of reaction for the CT beverage, at 15 min for the PD-SD and PD-FD beverages, and at 20 min for the IN-SD and IN-FD beverages. 

### 2.6. Sensory Analysis 

A total of 120 individuals were recruited for the sensory analysis. They were untrained individuals who were not allergic to dairy products and who liked dairy beverages. For the analysis, 25 mL of each labeled sample was presented randomly and monadically to the evaluators. Each evaluator received a card containing a nine-point hedonic scale (ranging from 1 = “dislike extremely” to 9 = “like extremely”) to evaluate the attributes appearance, aroma, body, flavor, and overall impression. Purchase intent was also assessed using a five-point scale (ranging from 1 = “definitely will not buy” to 5 = “definitely will buy”). The study was approved by the Ethics Committee for Research with Human Beings of the Federal University of Espírito Santo, Alegre-ES, Brazil (Protocol no. 2,669,719/2018).

### 2.7. In Vitro Gastrointestinal Digestion

In vitro simulated gastrointestinal digestion of the dairy beverages was performed in three replicates for each sample. A total of 15 mL sample was mixed in 30 mL of distilled water and the pH adjusted to 2.0 with HCl-added pepsin (0.04 g/g sample). The samples were kept in a shaking water bath at 37 °C for 2 h. After, the pH was adjusted to 7.0 with NaOH and pancreatin (0.14 g) and bile salts (0.87 g) were added. The samples were incubated at 37 °C for another 2 h. Hydrolysis was stopped in an ice bath for 10 min and the pH was adjusted to 2.0 to maintain the stability of the phenolic compounds. Samples were centrifuged (5400× *g*, 4 °C, 60 min) to separate the bioaccessible and the residual fractions [17]. Determination of levels caffeine, chlorogenic acid, and trigonelline in both fractions was performed following the method described in Section 2.3.4 above.

The recovery percentages of caffeine, chlorogenic acid, and trigonelline were determined in the bioaccessible fraction, residual fraction, and total recovery after in vitro simulated gastrointestinal digestion as:

- Bioacessible fraction (%) = (Qt supernatant/Qt digested) × 100
- Residual fraction (%) = (Qt residue/Qt digested) × 100
- Total recovery = bioaccessible fraction (%) + residual fraction (%)

Qt is the amount of caffeine, chlorogenic acid, or trigonelline.

Qt supernatant is the quantity of caffeine, chlorogenic acid, or trigonelline (mg) in the supernatant at the end of the corresponding phase of digestion. Qt residue is the quantity of caffeine, chlorogenic acid, or trigonelline (mg) in the pellet at the end of the corresponding phase of digestion and Qt digested is the quantity of caffeine, chlorogenic acid, or trigonelline (mg) that was submitted to digestion. Finally, total caffeine, chlorogenic acid, or trigonelline recovery during the steps of digestion was calculated.

### 2.8. Statistical Analysis

A completely randomized design was used in a factorial arrangement. The data were analyzed using one-way analysis of variance (ANOVA) followed by Tukey’s test to evaluate the differences between the treatments containing GCE and Dunnett’s test to compare the treatments with the control. The *t*-test was used to compare the content of total phenols and antioxidant activity before and after storage. A split-plot design was used for the study of stability during storage, in which the data were analyzed by ANOVA followed by regression analysis. The analyses were performed using the Statistica^®^ and GraphPad Prism software at a 5% significance level.

## 3. Results and Discussion

### 3.1. Characterization of the Encapsulated Extracts 

The extracts encapsulated by FD using IN as the encapsulating agent had significantly lower moisture content than those encapsulated by SD and those encapsulated with PD as the encapsulating agent (Table 1). Moisture is an important characteristic in powder products because it interferes with fluidity, stickiness, and stability during storage. The moisture values obtained in the different treatments were within an ideal range (below 5%), which ensures greater stability for powder products [18]. For A_w_, there was a significant difference between the encapsulation techniques, with the FD method providing encapsulated extracts with lower A_w_ (*p* < 0.05). Furthermore, the extracts encapsulated with IN had lower A_w_ (Table 1). These results can be associated with the vacuum effect, in which higher pressure gradients effect the water transfer rate, resulting in lower A_w_ in the final freeze-dried product [19].

Besides, the use of IN as encapsulating agent promoted a slight increase in pH (*p* < 0.05), compared to treatments with DP (Table 1). However, the values obtained remained within the parameters suitable for use in unfermented dairy beverages, which have pH values in the range of 5.0 to 6.8 [20]. 

Hygroscopicity is the ability of a material to absorb moisture from the environment and is an important property due to its effect on food stability [21]; materials that have values of hygroscopicity below 20% are considered less hygroscopic, which is a desirable characteristic [22]. The encapsulated extracts of this study had low hygroscopicity (Table 1) and the use of PD promoted an increase in hygroscopicity, especially when the FD encapsulation technique was used. This trend can be attributed to the higher hygroscopicity of PD, which tends to bind to water and form bridges between molecules [21]. According to Dias et al. [23] IN has fewer reducing groups and a higher glass transition temperature, with a lower tendency to absorb moisture from the environment. Another study also found that microcapsules produced with IN exhibited more desirable physical characteristics, such as lower values of moisture, Aw, and hygroscopicity [24]. As such, IN is an interesting carrier agent for the encapsulation of dry active compounds because lower water absorption values are desirable for better conservation and stability of the particles. 

The encapsulation techniques and agents used in the present study did not affect the samples’ solubility, content of total phenolic compounds, antioxidant activity, and content of caffeine and chlorogenic acid, because there was no significant difference among the treatments (*p* > 0.05). Regarding trigonelline content, there was no significant difference between the encapsulation techniques for the two encapsulating agents used (Table 1). However, when the SD technique was used, the extracts encapsulated with IN had lower trigonelline content. This is possibly explained by the interaction of trigonelline with IN. However, further studies are needed to determine the mechanisms of this interaction. 

**Figure 1:**
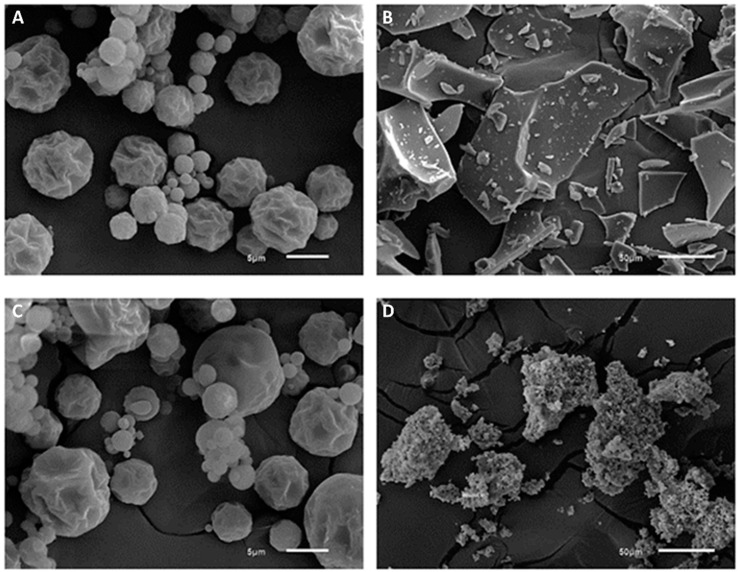
Micrographs of encapsulated green coffee extracts. (**A**) spray dryer with polydextrose; (**B**) spray dryer with inulin; (**C**) freeze-drying with polydextrose; (**D**) freeze-drying with inulin. (**A**,**B**) 3000× magnification; (**C**,**D**) 400× magnification.

The images obtained by SEM show that in the SD method the two encapsulating agents had similar particle morphology and size, with the diameter of the particles of extracts encapsulated with PD and IN ranging from 1 to 10 μm and 0.5 to 11 μm, respectively. The freeze-dried microparticles had a larger size, ranging from 11 to 183 μm in the extracts encapsulated with PD and from 5 to 103 μm in those encapsulated with IN (Figure 1). The values obtained are within the range expected for microparticles obtained by SD, whose diameter varies between 1 and 15 μm, whereas freeze-dried particles can reach 300 μm [25]. The smaller size of the particles obtained with the SD technique is due to the atomization of the liquid into small droplets during drying [26]. 

Most of the particles encapsulated by SD had a spherical shape with a wrinkled outer surface with some concavities, which are typical characteristics of spray-dried products [16,27]. The concavities present on the surface are likely formed by the shrinkage/contraction of the particles during the early stages of the drying process due to drastic moisture loss followed by cooling [16,24,25]. In addition, the wrinkled appearance of some particles may be related to the slower formation of the protective layer during the drying of the atomized droplets, and may be associated with the use of low drying temperatures. In this case, the formed protective layer remains moist and flexible for longer and the particle deflates and wrinkles while the water vapor within the particle condenses as the particle moves to the cooler areas of the dryer [23]. 

In contrast, the particles encapsulated by FD exhibited a morphology that varied from that of the particles dried by SD. According to Ballesteros et al. [27] the morphology of the capsules is expected to change after the FD and SD processes because of the different conditions that are used in each process. In the present study, the freeze-dried samples, regardless of the encapsulating agent, exhibited an irregular structure that resembled broken glass or sawdust and were of varying sizes. This morphology is typical of the FD process [25,27]. When comparing the freeze-dried samples, the structure of the particles of the freeze-dried extract encapsulated with IN was smaller and more irregular than that of the freeze-dried extract encapsulated with PD. The structural rigidity caused by the frozen surface and the lack of water in the liquid state results in a porous structure without shrinkage, which is the main characteristic of freeze-dried foods [25]. In addition, the larger particle size is attributed to the low temperature of the process and lack of pressure to break the frozen particles or to change the surface during drying [28]. 

### 3.2. Total Phenolics and Antioxidant Activity of Dairy Beverages during Storage

All beverages with added encapsulated GCEs differed from the CT beverage; they had a higher content of total phenolic compounds and higher antioxidant activity by the ABTS radical assay, both at the beginning and at the end of storage (Table 2). No significant differences were observed in these variables in relation to storage time, which indicates that there was no significant phenolic compound degradation during the study period. Moreover, at the end of storage, the beverages with encapsulated extract did not differ in total phenolic compounds and antioxidant activity by the ABTS assay. 

In contrast, the IN-SD beverage exhibited higher antioxidant activity than the CT beverage by the DPPH assay at the beginning of storage. However, there were no significant differences among the beverages with GCE (*p* > 0.05). Unlike what was observed in the ABTS radical assay, there was a decrease in the antioxidant activity of the dairy beverages at the end of storage (*p* ≤ 0.05). Furthermore, all beverages with added extracts had higher antioxidant activity than the CT beverage at the end of storage (*p* ≤ 0.05). Although there was a reduction in antioxidant content, the incorporation of GCEs was beneficial because the antioxidant potential of the product at the end of storage was higher than that of the control. 

The incorporation of encapsulated GCE provided an increase of up to 55% in the dairy beverages’ total phenolic content. This increase can be attributed to the presence of polyphenols and phenolic acids in the GCE, mainly chlorogenic acid [4]. The phenolic compounds found in the CT beverage are among the ingredients used in the formulation of dairy beverages, such as soluble coffee and chocolate powder. Similar results were obtained by Sęczyk & Świeca Michałand Gawlik-Dziki [29] who studied the inclusion of GCE in soy milk and observed an increase of up to 62% in the content of phenolic compounds, in addition to the improved antioxidant potential of the products. 

The assays with the ABTS and DPPH radicals resulted in some differences. The DPPH method provided lower Trolox values than the ABTS method due to the higher stability and lower reactivity of the DPPH radical, i.e., only strong reducing agents react with this radical. In addition, the DPPH radical reacts with polyphenols but not with phenolic acids or sugars. In contrast, the ABTS radical has the advantage of high reactivity and, therefore, the ability to react with a wide range of antioxidants [30]. Thus, the array of antioxidants determined by DPPH and ABTS is partially different. 

Moreover, phenolic compounds can interact with proteins, carbohydrates, lipids, and other phenolic compounds present in the food matrix, and these interactions influence their bioactivity [29]. Protein-phenolic interactions partially mask the anti-radical activity of phenolic compounds by blocking their reactive groups [31]. Theoretically, these changes in the structure of antioxidant compounds affect their reaction with radicals, potentially leading to an underestimation of the total antioxidant capacity of the product [32]. 

### 3.3. Bioactive Compounds in Dairy Beverages during Storage

The main challenge in the production of encapsulated materials consists in obtaining products with desirable characteristics, at reduced costs, that remain stable during storage [18]. However, it is also important to evaluate the behavior of the encapsulated components once incorporated into a food matrix to monitor possible changes and ensure the stability of bioactive compounds during storage. 

**Figure 2:**
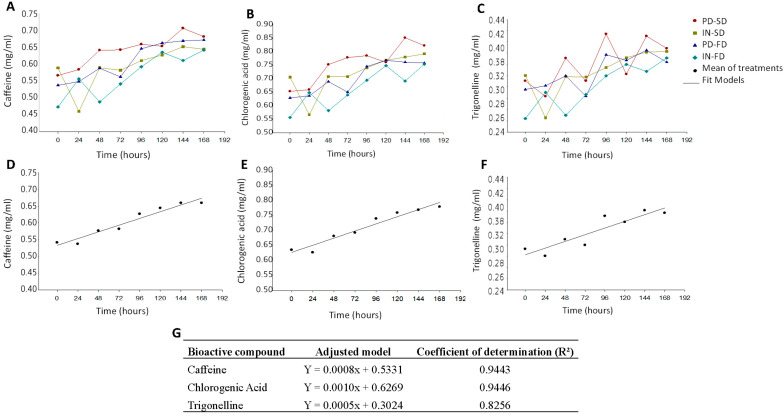
Content of caffeine, chlorogenic acid, and trigonelline during storage in dairy beverages incorporated with encapsulated extracts of green coffee. (**A**) Dispersion of the caffeine content in each beverage incorporated; (**B**) dispersion of the chlorogenic acid content in each beverage incorporated; (**C**) dispersion of trigonelline content in each beverage incorporated; (**D**) general behavior of caffeine for all beverages; (**E**) general behavior of chlorogenic acid for all beverages; (**F**) general behavior of trigonelline for all beverages; (**G**) models fitted for caffeine, chlorogenic acid, and trigonelline. PD-SD: beverage added from the extract encapsulated with polydextrose by spray-dryer; IN-SD: beverage added from the extract encapsulated with inulin by spray-dryer; PD-FD: beverage added from the extract encapsulated with polydextrose by freeze-drying; IN-FD: beverage added from the extract encapsulated with inulin by freeze-drying.

The concentrations of caffeine, chlorogenic acid, and trigonelline did not differ among the treatments (Figure 2A–C); however, changes occurred with storage duration that the content of caffeine increased from 0.46 to 0.71 mg/mL (Figure 2A), the chlorogenic acid increased from 0.53 to 0.85 mg/mL (Figure 2B), and that of trigonelline increased from 0.26 to 0.41 mg/mL (Figure 2C). 

To better understand the behavior of each bioactive compound during storage, regression analysis was performed using the means of the treatments (Figure 2D–F). The mathematical model used to describe the behavior of each bioactive compound and their determination coefficient value (R²) was the linear polynomial model, with demonstrated that samples had satisfactory R² values (above 0.8) (Figure 2G). These results show that the bioactive compounds had similar behaviors, namely an increase in concentration in the dairy beverages over the seven days of storage. This fact can be attributed to the microencapsulated materials, which control the release of the active material over time. Through this mechanism, the encapsulated bioactive compounds are gradually released into the medium in which they are dispersed, which promotes the maintenance of these compounds in the food matrix [33]. The controlled release profile was studied by Campelo-Felix et al. [34] in lime essential oil microparticles using oligofructose and IN as wall material. The results showed that the microparticles containing the prebiotic polymers in their composition allowed a constant (linear) release rate of the essential oil. 

The stability of chlorogenic acid can be affected when it is exposed to high temperatures or alkaline conditions, and it can be isomerized, hydrolyzed, or decomposed to low-molecular weight compounds [35]. The same occurs for trigonelline, which is an alkaloid that is thermally unstable and undergoes degradation at high temperatures [36,37]; caffeine is stable at high temperatures [37]. It is noteworthy that the conditions used in the present study for the preparation and storage of the dairy beverages with added encapsulated extracts did not involve high temperatures or pH values that could affect the stability of these bioactive compounds and their content in the formulated product. 

Another factor that may have contributed to the increase in the content of bioactive compounds over time is that the extraction method was effective in removing caffeine, chlorogenic acid, and trigonelline that were solubilized in them. However, it was not effective in extracting the bioactive compounds that were still protected by the microcapsules. Thus, further studies are needed to evaluate the efficacy of different methods of extraction of these compounds. 

### 3.4. Percentage of Recovery after In Vitro Gastrointestinal Digestion 

**Figure 3:**
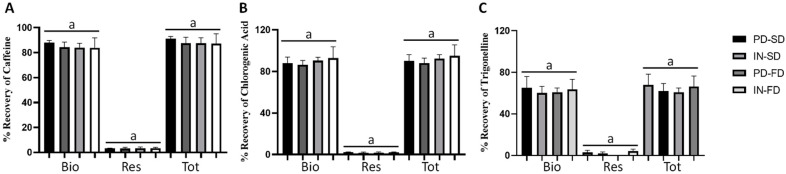
Percentage recovery of main phenolic compounds presents in beverages incorporated with encapsulated extracts of green coffee after in vitro digestion. (**A**) Caffeine; (**B**) chlorogenic acid; (**C**) trigonelline. Bio: bioaccessible fraction; Res: residual fraction; Tot: % Total Recovery. Data presented as mean ± standard deviation of three replicates. Different lower-case letters in each sample represent statistical difference according to the Tukey test (*p* ≤ 0.05). PD-SD: beverage added from the extract encapsulated with polydextrose by spray-dryer; IN-SD: beverage added from the extract encapsulated with inulin by spray-dryer; PD-FD: beverage added from the extract encapsulated with polydextrose by freeze-drying; IN-FD: beverage added from the extract encapsulated with inulin by freeze-drying.

After simulated in vitro gastrointestinal digestion, it was observed that the contents of the main phenolic compounds of the green coffee extract, caffeine, chlorogenic acid, and trigonelline were similar in all samples, both in the bioaccessible fraction, in the residue, and the total percentage of recovery (Figure 3). However, it was observed that the values were around 90%, which is considered a good percentage of recovery and supports the idea that in vitro digestion releases large amounts of phenolic and antioxidant compounds [31]. 

The decrease in phenolic compounds after digestion could be related to their transformation into different structural forms during digestion, especially after the intestinal phase, since phenolic compounds are highly sensitive to alkaline conditions [38], then this can explain the reduction of phenolic compounds, after the in vitro gastrointestinal digestion, in this study.

However, the residual fraction after digestion is not considered when studying phenolic bioaccessibility because large amounts of phenolic compounds usually escape absorption in the small intestine. These unabsorbed phenolic compounds enter the large intestine where they exert a prebiotic effect by interacting with the microbiota, even mucosal cells, and dendritic projections in the lumen, also exerting antioxidant effects in the gastrointestinal tract [31,39]. 

This gastrointestinal in vitro digestion technic is a suitable, inexpensive, and simple alternative to assess the bioaccessibility of compounds from the food matrix under gastrointestinal conditions; however, many methodologies use different conditions, which affects the results, making a comparison between studies very difficult. However, our results are superior to the previous study with microencapsulated phenolic compounds of jussara (*Euterpe edulis Martius*) fruit [17] and tartary buckwheat (*Fagopyrum tataricum Gaertn*.) [32].

### 3.5. Sensory Analysis of Dairy Beverages 

The effect of incorporating encapsulated GCE in dairy beverages on the sensory attributes of the product showed that the acceptance of the appearance attribute was significantly lower (*p* ≤ 0.05) for the dairy beverages with added green coffee extract than for the CT beverage (Table 3). There were no significant differences in the aroma and body attributes (*p* > 0.05). The statistical analysis comparing the samples with green coffee extract showed that the different techniques and agents used for encapsulation did not affect the appearance, aroma, or body attributes of the products (Table 3). 

Regarding the perception of flavor, overall impression, and purchase intent, the dairy beverages with added green coffee extracts encapsulated by FD did not differ from the CT beverage (*p* > 0.05). On the contrary, beverages with extracts encapsulated by SD received significantly lower scores (*p* ≤ 0.05) of these attributes than the CT beverage. Moreover, the comparison of the different techniques and encapsulating materials showed that the samples with freeze-dried green coffee extracts had higher scores for flavor (*p* ≤ 0.05) than those with extracts encapsulated by SD. The IN-FD beverage had significantly higher scores for overall impression and purchase intent than the beverages with extracts encapsulated by SD. 

In addition to the nutritional properties of beverages with added green coffee and the functional properties of dietary fibers (IN and PD), sensory attributes are important factors for consumer acceptance [29]. Based on the sensory scale scores, all tested products showed good sensory acceptance, with the mean scores of all attributes varying between the hedonic terms “liked moderately” to “liked very much”. However, dairy beverages with added freeze-dried extracts had higher acceptance scores regarding flavor, overall impression, and purchase intent, with the IN-FD beverage showing higher acceptance for the last two attributes compared to beverages with added extracts encapsulated by SD. 

Therefore, given the sensory acceptance of beverages with microencapsulated extracts, as well as the high antioxidant activity, quantity and bioaccessibility of phenolic compounds and stability of these compounds observed in this study, these extracts can form an aid strategy against non-communicable and chronic diseases, mainly insulin resistance, cardiovascular diseases, cancer, etc., due to the ability to scavenge free radicals, preventing them from acting negatively on metabolic pathways and DNA damage [40,41]. Thus, given the growing interest of the population in products with health benefits, including antioxidant foods [42], these developed extracts can add value to the food products and health to the population.

As limitations of this study and suggestion for future studies, we can mention the lack of analysis of the content of total phenolics and antioxidant activity of the extracts between the times of 0 and 168 h, as well as to evaluate the profile of the phenolics present in both extracts. In addition, the stability and concentration of the compounds could be evaluated for a period longer than 1 week and the microencapulated extracts could have been added to fermented dairy drinks to verify whether fermentation modifies the evaluated markers.

## 4. Conclusions

The use of the SD and FD techniques, together with the use of PD and IN, was effective in the microencapsulation of GCE. The extracts encapsulated with IN by FD exhibited more desirable physical characteristics, such as lower moisture, water activity, and hygroscopicity. The addition of the encapsulated green coffee extracts to unfermented dairy beverages resulted in an increase in the content of phenolic compounds and the antioxidant properties of the products. Moreover, microencapsulation allowed the controlled release of the bioactive compounds throughout the storage period and every microencapsulation technique demonstrated similar bioaccessibility after gastrointestinal digestion. The dairy beverages had good acceptance among consumers, with the IN-FD beverage having the best acceptance scores for overall impression and purchase intent. Thus, microencapsulation of green coffee extracts was shown to be a promising technique for obtaining bioactive compounds, with potential application in the production of foods with functional properties.

## Figures and Tables

**Table 1:** Effect of encapsulation techniques and wall materials on the characteristics of the encapsulated extracts.

Analysis	Encapsulation Technique	Encapsulating Agent	
		PD	IN
Moisture (g/100 g)	SD	4.19 ± 0.26 ^aA^	4.22 ± 0.48 ^aA^
	FD	3.30 ± 0.69 ^aA^	0.35 ± 0.03 ^bB^
Water activity	SD	0.35 ± 0.03 ^aA^	0.33 ± 0.01 ^aA^
	FD	0.16 ± 0.04 ^bA^	0.03 ± 0.001 ^bB^
pH	SD	5.34 ± 0.07 ^aA^	5.76 ± 0.09 ^aB^
	FD	5.27 ± 0.04 ^aA^	5.75 ± 0.08 ^aB^
Hygroscopicity (%)	SD	18.40 ± 0.34 ^aA^	14.55 ± 0.46 ^aB^
	FD	19.91 ± 0.52 ^bA^	14.92 ± 0.97 ^aB^
Solubility (%)	SD	97.23 ± 2.23 ^aA^	98.42 ± 1.66 ^aA^
	FD	97.79 ± 1.70 ^aA^	96.76 ± 3.55 ^aA^
Total Phenolic Compounds (mg GAE/g)	SD	19.01 ± 5.96 ^aA^	21.08 ± 2.97 ^aA^
	FD	22.43 ± 2.77 ^aA^	21.17 ± 1.59 ^aA^
Antioxidant activity (ABTS) (µmol Trolox/g)	SD	153.96 ± 16.93 ^aA^	143.53 ± 13.71 ^aA^
	FD	154.76 ± 28.94 ^aA^	166.45 ± 30.35 ^aA^
Antioxidant activity (DPPH) (µmol Trolox/g)	SD	110.85 ± 24.89 ^aA^	105.03 ± 22.91 ^aA^
	FD	88.45 ± 13.77 ^aA^	97.95 ± 19.07 ^aA^
Caffeine (mg/g)	SD	9.59 ± 0.91 ^aA^	9.67 ± 0.51 ^aA^
	FD	9.22 ± 0.31 ^aA^	9.29 ± 0.25 ^aA^
Chlorogenic acid (mg/g)	SD	18.71 ± 1.79 ^aA^	18.60 ± 0.98 ^aA^
	FD	17.79 ± 0.50 ^aA^	17.88 ± 0.50 ^aA^
Trigonelline (mg/g)	SD	5.73 ± 0.45 ^aA^	4.87 ± 0.41 ^aB^
	FD	5.31 ± 0.16 ^aA^	4.55 ± 0.38 ^aA^

Values expressed as mean ± standard deviation (No repetitions = 4). Means followed by different lower-case letters in the same column of an analysis differ significantly (*p* ≤ 0.05). Means followed by different capital letters in the same row of an analysis differ significantly (*p* ≤ 0.05). SD: Spray drying; FD: Freeze-drying; PD: Polydextrose; IN: Inulin; GAE: gallic acid equivalent.

**Table 2:** Total phenolic content and antioxidant activity of the prepared beverages, at the beginning (T 0 h) and at the end (T 168 h) of storage.

	Samples	Storage Time		
		T 0 h	T 168 h	% of Remaining
Total phenolic compounds (mg GAE/mL)	CT	2.20 ± 0.04	2.41 ± 0.07	108.79 ± 1.74
	PD-SD	3.42 ± 0.02 * ^a.A^	3.25 ± 0.10 * ^a.A^	90.29 ± 8.09 * ^a^
	IN-SD	3.27 ± 0.10 * ^ab.A^	3.20 ± 0.14 * ^a.A^	94.24 ± 2.31 * ^a^
	PD-FD	3.30± 0.12 * ^ab.A^	3.15 ± 0.04 * ^a.A^	92.34 ± 3.12 * ^a^
	IN-FD	3.00± 0.08 * ^b.A^	3.05 ± 0.07 * ^a.A^	96.71 ± 6.38 ^a^
Antioxidant activity (ABTS) (µmol Trolox/mL)	CT	84.59 ± 5.07	85.35 ± 2.31	98.91 ± 2.02
	PD-SD	102.90 ± 4.03 * ^a.A^	101.25 ± 1.35 * ^a.A^	93.47 ± 2.29 ^a^
	IN-SD	103.78 ± 6.00 * ^a.A^	104.43 ± 1.30 * ^a.A^	97.09 ± 5.58 ^a^
	PD-FD	105.41 ± 2.41 * ^a.A^	109.30 ± 3.90 * ^a.A^	100.24 ± 2.59 ^a^
	IN-FD	105.33 ± 4.79 * ^a.A^	107.62 ± 0.95 * ^a.A^	99.77 ± 6.93 ^a^
Antioxidant activity (DPPH) (µmol Trolox/mL)	CT	11.31 ± 0.66	2.82 ± 1.51	24.72 ± 10.72
	PD-SD	12.89 ± 0.35 ^a.A^	9.01 ± 1.12 * ^a.B^	66.23 ± 3.43* ^a^
	IN-SD	14.56 ± 0.58 * ^a.A^	6.7 ± 0.37 * ^a.B^	44.92 ± 3.74 ^a^
	PD-FD	14.02 ± 0.83 ^a.A^	7.77 ± 1.22 * ^a.B^	53.87 ± 11.42 ^a^
	IN-FD	13.53 ± 0.80 ^a.A^	8.13 ± 2.94 * ^a.B^	57.87 ± 149.59 ^a^

Values expressed as mean ± standard deviation. GAE: gallic acid equivalent. CT: control; PD-SD: beverage with added extract encapsulated with polydextrose by spray drying; IN-SD: beverage with added extract encapsulated with inulin by spray drying; PD-FD: beverage with added extract encapsulated with polydextrose by freeze-drying; IN-FD: beverage with added extract encapsulated with inulin by freeze-drying. * indicates a significant difference between the control and the samples according to Dunnett’s test. GAE: Gallic acid equivalent. Means followed by different lower-case letters in the same column of a variable are significantly different according to Tukey’s test (*p* ≤ 0.05). Means followed by different capital letters in the same row of a variable are significantly different according to the *t*-test (*p* ≤ 0.05).

**Table 3:** Means of the hedonic scores obtained in the sensory analysis of the dairy beverages with added green coffee.

Samples	Attributes					
	Appearance	Aroma	Body	Flavor	Overall Impression	Purchase Intent
CT	7.97	7.30	7.26	7.68	7.58	4.04
PD-SD	7.20 *^a^	7.24 ^a^	7.11 ^a^	7.32 *^a^	7.28 *^a^	3.74 *^a^
IN-SD	7.31 *^a^	7.05 ^a^	7.26 ^a^	7.30 *^a^	7.31 *^a^	3.74 *^a^
PD-FD	7.16 *^a^	7.18 ^a^	7.26 ^a^	7.76 ^b^	7.43 ^ab^	3.93 ^ab^
IN-FD	7.31 *^a^	7.26 ^a^	7.23 ^a^	7.81 ^b^	7.64 ^b^	3.97 ^b^

CT: control; PD-SD: beverage with added extract encapsulated with polydextrose by spray drying; IN-SD: beverage with added extract encapsulated with inulin by spray drying; PD-FD: beverage with added extract encapsulated with polydextrose by freeze-drying; IN-FD: beverage with added extract encapsulated with inulin by freeze-drying. * indicates a significant difference between the control and the samples according to Dunnett’s test. Means followed by different lower-case letters in the same column of a variable are significantly different according to Tukey’s test (*p* ≤ 0.05).

## Data Availability

Not applicable.
